# Preoperative risk score for malignancy in Bethesda III thyroid nodules: a multicentre retrospective study

**DOI:** 10.3389/fendo.2026.1863739

**Published:** 2026-07-06

**Authors:** Manuel Carpio-Salmerón, Mariano Tébar-Caballero, Pau Benito, Clemente García-Hidalgo, Georgios Kyriakos, Antonio Ríos-Vergara, Ginés Luengo-Gil, Carlos Carazo-Casas, Ana Casas-Miras, Luis Marín-Martínez, Ana Belén Arroyo

**Affiliations:** 1 Department of Endocrinology and Nutrition, Santa Lucía University General Hospital, Cartagena, Spain; 2 Department of Internal Medicine, Reina Sofía Hospital, Murcia, Spain; 3 Department of Preventive Medicine and Epidemiology, Clinical Institute of Medicine and Dermatology (ICMiD), Hospital Clínic de Barcelona, Barcelona, Spain; 4 Department of Radiology, Hospital Morales Meseguer, Murcia, Spain; 5 Group of Molecular Pathology and Pharmacogenetics, Pathology Department, IMIB-Pascual Parrilla, Hospital General Universitario Santa Lucía, Cartagena, Spain; 6 Health Sciences Faculty, Universidad Católica de Murcia (UCAM), Guadalupe, Murcia, Spain; 7 Department of Otolaryngology, Ramón y Cajal Hospital, Madrid, Spain; 8 General University Hospital Santa Lucía, Pathological Anatomy, Cartagena, Spain

**Keywords:** ACR-TIRADS, atypia of undetermined significance, Bethesda III, lymphocytic thyroiditis, malignancy prediction, nuclear atypia, risk score

## Abstract

**Introduction:**

Bethesda III thyroid nodules represent a heterogeneous cytological category with an estimated malignancy risk of 13–30%, creating uncertainty in clinical decision-making. This study aimed to characterise surgically resected Bethesda III thyroid nodules in a multicentre cohort and to develop a simple preoperative risk score for malignancy prediction using routinely available variables.

**Methods:**

We performed a retrospective, observational, multicentre study across four tertiary hospitals in the Region of Murcia, Spain. Adults with Bethesda III thyroid nodules who underwent thyroidectomy between 2020 and 2025 were included. Clinical, biochemical, ultrasonographic, cytological, and histopathological variables were analysed using univariate and multivariable logistic regression. A simplified integer-based risk score was derived from the independent predictors identified in the final model.

**Results:**

Eighty-five nodules were included, with a malignancy rate of 23.5%. Three preoperative variables remained independently associated with malignancy: moderate-to-high ultrasound risk, nuclear atypia on fine-needle aspiration, and lymphocytic thyroiditis on fine-needle aspiration. These predictors were incorporated into a practical score ranging from 0 to 15 points. The model showed acceptable apparent discrimination and calibration, although internal bootstrap validation indicated that cautious interpretation is required because of the limited sample size. A cut-off of 10 points or higher provided the best overall diagnostic performance, and the predicted probability of malignancy ranged from 2.4% to 74.8% across the score spectrum.

**Discussion:**

In this multicentre cohort of surgically resected Bethesda III thyroid nodules, a simple three-item exploratory score showed promising performance for preoperative malignancy risk stratification. This tool may help support clinical decision-making using variables available in routine practice, although external validation in larger independent cohorts is required before routine clinical implementation.

## Introduction

Thyroid nodules are focal lesions within the thyroid parenchyma that can be detected by palpation or imaging. Their prevalence in the general population is high, reaching 40% to 67% in ultrasonographic and autopsy studies, although only 3% to 7% of them are clinically palpable (1, 2). Despite this high frequency, the overall probability of malignancy is relatively low, estimated at 4%-6.5%, and the majority of nodules are benign (3).

Given the high prevalence of thyroid nodules and the risk of overdiagnosis and overtreatment, contemporary management aims to provide the least invasive intervention compatible with oncologic safety. Current risk-adapted approaches emphasize integrating clinical context, ultrasound findings, cytology, and patient preferences in order to reduce unnecessary procedures and diagnostic surgery while maintaining adequate cancer detection (4, 5).

The prevalence of thyroid nodules is higher in women and increases with age, particularly after the fifth decade of life (6). Furthermore, the incidence is elevated in iodine-deficient regions and in patients with autoimmune thyroiditis, such as Hashimoto’s disease, in which chronic inflammation and reactive hyperplasia promote nodule formation (7). Risk factors for the development of malignant neoplasms include genetic mutations in BRAF, RAS, TERT, and TP53; exposure to ionising radiation; elevated TSH levels; a family history of thyroid cancer; and hereditary syndromes such as MEN2 or Cowden syndrome (1, 6). Environmental and metabolic factors, including obesity, smoking, and exposure to toxins, may also contribute to the development of malignant lesions (8).

The primary objective of thyroid nodule evaluation is to exclude malignancy (1). Thyroid ultrasound is a fundamental tool in the initial assessment, allowing the characterisation of morphology, composition, echogenicity, margins, and the presence of micro- or macrocalcifications. These sonographic features are critical for estimating the risk of malignancy and selecting nodules that warrant cytological evaluation by fine needle aspiration (FNA) (9).

To standardise this assessment and reduce inter-observer variability, the American College of Radiology developed the Thyroid Imaging Reporting and Data System (ACR-TIRADS), which assigns a quantitative score based on five key sonographic features: composition, echogenicity, shape, margins, and echogenic foci (9). Each feature contributes a specific score, and the total score determines the risk category, ranging from TIRADS 1 (benign) to TIRADS 5 (highly suspicious for malignancy). Higher categories are associated with progressively increasing malignancy risk, from less than 2% in TIRADS 1–2 to more than 20% in TIRADS 5, and guide the indication for surveillance or FNA biopsy (9, 10).

Once sonographic risk is established, nodules meeting suspicion criteria undergo FNA, a technique with high sensitivity, low cost, and minimal invasiveness, and is considered the reference standard for the cytological diagnosis of thyroid lesions (1, 11). FNA results were interpreted according to the Bethesda System for Reporting Thyroid Cytopathology (2023 edition), which classifies specimens into six categories with corresponding malignancy risks and direct clinical recommendations (12).

Bethesda category III includes nodules with atypia of undetermined significance (AUS). In the 2023 revision, the previously used term follicular lesion of undetermined significance (FLUS) was grouped under AUS-other. Overall, these nodules carry a ROM between 13% and 30%, generating a management dilemma and diagnostic challenge, as this category groups lesions with inconclusive cytological abnormalities that do not allow definitive differentiation between benign and malignant nodules (11, 12). This heterogeneity is clinically relevant because nodules with nuclear atypia consistently show a higher malignancy risk than those with predominantly architectural atypia. This distinction has been supported not only by institutional series but also by higher-level evidence, including a systematic review and meta-analysis and a Spanish cohort showing substantially higher malignancy rates in the cytological atypia subgroup (13–17). The incidence of this category has increased in parallel with the wider use of high-resolution ultrasonographic techniques, and its management remains controversial, as a substantial proportion of patients undergo surgery (hemi- or total thyroidectomy), with a large fraction of lesions proving benign on final pathology, leading to unnecessary overtreatment and permanent complications (18, 19).

We hypothesised that the systematic integration of clinical, cytological, and ultrasonographic variables would enable the establishment of risk subcategories within Bethesda III, identifying a high-risk subgroup. These basic diagnostic features, routinely available in most hospital settings, could serve as a first-line assessment tool to rationalise the indication for repeat FNA, molecular testing, or diagnostic-therapeutic surgery. Accurate identification of these risk patterns is key to minimising both unnecessary surgical overtreatment and diagnostic delays in malignant lesions.

Several strategies have been proposed to refine malignancy risk in Bethesda III thyroid nodules, including cytological subcategorization according to nuclear atypia, repeat fine-needle aspiration combined with ultrasound risk stratification, critical ultrasound reassessment, and molecular testing. However, there remains a need for simple preoperative tools derived in multicentre cohorts and based on routinely available variables that integrate ultrasound, cytology, and inflammatory context, particularly in settings where molecular testing is not universally accessible (13, 20, 21).

This present study aimed to derive an exploratory preoperative multivariable model and a simplified risk score for malignancy stratification in surgically resected Bethesda III thyroid nodules using routinely available clinical, cytological, and ultrasonographic variables. The secondary aims were to characterise the clinical, laboratory, ultrasonographic, cytological, and histopathological features of the cohort, estimate the malignancy rate in this surgically selected population, and assess the discriminatory performance of the derived score in the derivation cohort.

## Materials and methods

Study design. This was a retrospective, observational, and multicentre study. Consecutive patients diagnosed with Bethesda III thyroid nodules who underwent surgical resection were identified at four tertiary hospitals in the Region of Murcia, Spain. The inclusion period spanned from 2020 to 2025. Data were collected retrospectively from electronic medical records, radiology reports, cytology reports, and surgical pathology reports. All Bethesda III FNA specimens were re-reviewed by dedicated head and neck pathologists at each participating hospital, all of whom had subspecialty expertise in thyroid cytopathology. Because the original cytological slides are archived indefinitely in the participating institutions, a complete and systematic reclassification into nuclear atypia versus other patterns (according to the 2023 Bethesda System) was performed in every case. Lymphocytic thyroiditis on FNA was defined as the presence of a moderate-to-marked lymphoid infiltrate (≥10 lymphocytes per high-power field or lymphoid aggregates with germinal centres) in the cytological smear, according to the Bethesda System 2023 criteria and Cho et al. (12, 22). The presence of lymphocytic thyroiditis was recorded based on the cytological assessment of the FNA specimen.

Study population. The study population comprised patients managed in the endocrinology and/or General Surgery departments of the participating hospitals who had thyroid nodules classified as Bethesda III on FNA and subsequently underwent thyroid surgery.

The inclusion criteria were as follows: age ≥18 years; Bethesda III thyroid nodule; thyroid surgery performed between 2020 and 2025; availability of an evaluable thyroid ultrasound report allowing ACR-TIRADS classification; and availability of a definitive histological report of the surgical specimen.

The exclusion criteria were as follows: patients with Bethesda III nodules who did not undergo surgery, patients without sufficient ultrasonographic data for ACR-TIRADS classification and medical records with incomplete information on critical variables for the primary analysis.

Statistical analysis. Categorical variables were expressed as frequencies and percentages. Associations between clinical, ultrasonographic, and cytological features and malignancy status (benign vs. malignant on final histology) were assessed using the chi-square test or Fisher’s exact test, as appropriate. ACR-TIRADS was dichotomised as low risk (TR 1-3) versus moderate-high risk (TR 4-5). Crude odds ratios (OR) with 95% confidence intervals (CI) were calculated. Variables with a *p*-value<0.05 in the univariate analysis were considered candidates for the multivariable logistic regression model. The final exploratory multivariable logistic regression model was constructed using backward stepwise elimination of candidate predictors. A simplified integer-based risk score was derived from the beta coefficients of the final multivariable model. To preserve the relative contribution of each predictor while enhancing bedside usability, beta coefficients were converted into an integer score proportional to their magnitude, yielding 6 points for moderate-to high-risk ACR-TIRADS (TR 4-5), 5 points for nuclear atypia on FNA, and 4 points for lymphocytic thyroiditis on FNA. This approach was intended to provide a clinically practical approximation of the underlying regression model while retaining the relative weights of the independent predictors. The predictive performance of the score was evaluated across multiple cutoff points by calculating the sensitivity, specificity, and Youden Index. The discriminative ability of the score was assessed using receiver operating characteristic (ROC) curve analysis, and the area under the ROC curve (AUC) with 95% confidence intervals was calculated. The optimal cut-off was defined as the score maximizing the Youden Index. A two-sided *p-*value of <0.05 was considered statistically significant. Given the limited number of malignant events, the model complexity was intentionally restricted to reduce overfitting. Missing data were low and are presented in **Table 1**. Analyses were performed using the available case data.

**Table 1:** Clinical, cytological, and sonographic features of benign and malignant Bethesda III thyroid nodules. Values are n (%). Statistically significant results are highlighted.

Variable	All (n=85)	Benign (n=65)	Malignant (n=20)	Crude OR (95% CI)	P-value
Sociodemographic variables					
Sex: Male	18 (21.2)	12 (18.5)	6 (30.0)	1.89 (0.57-5.85)	0.274
Sex: Female	67 (78.8)	53 (81.5)	14 (70.0)	Ref	
Age <45	23 (27.1)	19 (29.2)	4 (20.0)	Ref	
Age 45-54	25 (29.4)	15 (23.1)	10 (50.0)	3.17 (0.87-13.45)	0.09
Age ≥55	37 (43.5)	31 (47.7)	6 (30.0)	0.92 (0.23-3.99)	0.91
Smoking: Yes	33 (38.8)	27 (41.5)	6 (30.0)	0.60 (0.19-1.71)	0.357
Alcohol intake: Yes	11 (12.9)	10 (15.4)	1 (5.0)	0.29 (0.02-1.67)	0.252
Clinical variables					
Normal weight	26 (30.6)	21 (32.3)	5 (25.0)	Ref	
Overweight	32 (37.6)	22 (33.8)	10 (50.0)	1.91 (0.58-7.00)	0.302
Grade 1 obesity	17 (20.0)	14 (21.5)	3 (15.0)	0.90 (0.16-4.29)	0.896
Grade 2 obesity	4 (4.7)	3 (4.6)	1 (5.0)	1.40 (0.06-13.97)	0.789
Grade 3 obesity	4 (4.7)	3 (4.6)	1 (5.0)	1.40 (0.06-13.97)	0.789
Dyslipidemia: Yes	40 (47.1)	29 (44.6)	11 (55.0)	1.52 (0.55-4.24)	0.417
Type 2 diabetes: Yes	16 (18.8)	11 (16.9)	5 (25.0)	1.64 (0.46-5.29)	0.422
Prior thyroid nodules: Yes	57 (67.1)	46 (70.8)	11 (55.0)	0.50 (0.18-1.44)	0.194
Prior thyroid cancer: Yes	3 (3.5)	3 (4.6)	0 (0)	NA	NA
Prior neck radiation: Yes	2 (2.4)	2 (3.1)	0 (0)	NA	NA
MEN: Yes	1 (1.2)	1 (1.5)	0 (0)	NA	NA
Levothyroxine: Yes	13 (15.3)	10 (15.4)	3 (15.0)	0.97 (0.20-3.61)	0.967
Blood tests					
TSH: ≤0.27 mUI/L	8 (9.4)	8 (12.3)	0 (0)	NA	NA
TSH: 0.28-4.19 mUI/L	63 (74.1)	47 (72.3)	16 (80.0)	Ref	
TSH: >4.20 mUI/L	14 (16.5)	10 (15.4)	4 (20.0)	1.18 (0.29-4.08)	0.807
Anti-TPO: Yes	15 (17.6)	9 (13.8)	6 (30.0)	2.51 (0.73-8.34)	0.132
Anti-TG: Yes	7 (8.2)	5 (7.7)	2 (10.0)	1.25 (0.17-6.39)	0.802
Ultrasound findings					
Cystic or spongiform	4 (4.7)	4 (6.2)	0 (0)	NA	NA
Mixed cystic-solid	6 (7.1)	5 (7.7)	1 (5.0)	0.59 (0.03-3.97)	0.64
Solid	75 (88.2)	56 (86.2)	19 (95.0)	Ref	
Anechoic	4 (4.7)	4 (6.2)	0 (0)	NA	NA
Markedly hypoechoic	3 (3.5)	1 (1.5)	2 (10.0)	6.00 (0.52-137.56)	0.16
Hypoechoic	38 (44.7)	30 (46.2)	8 (40.0)	0.80 (0.27-2.30)	0.68
Hyperechoic or isoechoic	40 (47.1)	30 (46.2)	10 (50.0)	Ref	
No echogenic focus	63 (74.1)	54 (83.1)	9 (45.0)	Ref	
Macrocalcifications	16 (18.8)	9 (13.8)	7 (35.0)	4.67 (1.37-16.04)	0.01
Microcalcifications	6 (7.1)	2 (3.1)	4 (20.0)	12.00 (2.04-96.47)	<0.01
Shape: Ovoid	75 (88.2)	60 (92.3)	15 (75.0)	Ref	
Shape: Taller-than-wide	10 (11.8)	5 (7.7)	5 (25.0)	4.00 (1.00-16.20)	0.046
Margins: Well/poorly defined	80 (94.1)	64 (98.5)	16 (80.0)	Ref	
Margins: Lobulated/irregular	5 (5.9)	1 (1.5)	4 (20.0)	16.00 (2.19-324.78)	0.02
TR 1	4 (4.7)	4 (6.2)	0 (0)	NA	NA
TR 2	2 (2.4)	2 (3.1)	0 (0)	NA	NA
TR 3	27 (31.8)	25 (38.5)	2 (10.0)	Ref	
TR 4	40 (47.1)	30 (46.2)	10 (50.0)	4.17 (0.98-28.78)	0.08
TR 5	12 (14.1)	4 (6.2)	8 (40.0)	25.00 (4.49-215.15)	<0.01
Low risk (TR 1-3)	33 (38.8)	31 (47.7)	2 (10.0)	Ref	
Moderate-high risk (TR 4-5)	52 (61.2)	34 (52.3)	18 (90.0)	8.21 (2.13-54.30)	<0.01
Size ≤20 mm	23 (27.1)	14 (21.5)	9 (45.0)	2.98 (1.02-8.71)	0.04
Size >20 mm	62 (72.9)	51 (78.5)	11 (55.0)	Ref	
Nuclear atypia on FNA: Yes	27 (31.8)	14 (21.5)	13 (65.0)	6.37 (2.19-20.02)	<0.01
Nuclear atypia on FNA: No	55 (64.7)	48 (73.8)	7 (35.0)	Ref	
Lymphocytic thyroiditis on FNA: Yes	30 (35.3)	18 (27.7)	12 (60.0)	3.83 (1.37-11.33)	0.01
Lymphocytic thyroiditis on FNA: No	54 (63.5)	46 (70.8)	8 (40.0)	Ref	

OR, odds ratio; CI, confidence interval; FNA, fine-needle aspiration; TIRADS, Thyroid Imaging Reporting and Data System; MEN, Multiple Endocrine Neoplasia; NA, not applicable.

Internal validation of the risk score and underlying logistic regression model was performed using bootstrap resampling with 1,000 replications. Discrimination was assessed using the area under the ROC curve (AUC), and the optimism-corrected AUC was calculated. Model calibration was evaluated using the optimism-corrected calibration slope and intercept, and a calibration plot was generated by plotting the observed versus predicted probabilities.

This multicentre retrospective observational cohort study was conducted in accordance with the principles of the Declaration of Helsinki. The study protocol was reviewed and approved by the local Research Ethics Committee of Santa Lucía University General Hospital (Cartagena, Spain), which is the coordinating centre. Data from the other three participating hospitals were obtained after formal data transfer and cession agreements were signed by the respective hospitals. All patient data were fully anonymised before collection, pooling and analysis. Because of the retrospective design and exclusive use of de-identified data, the requirement for individual informed consent was waived by the Ethics Committee.

## Results

**Figure 1:**
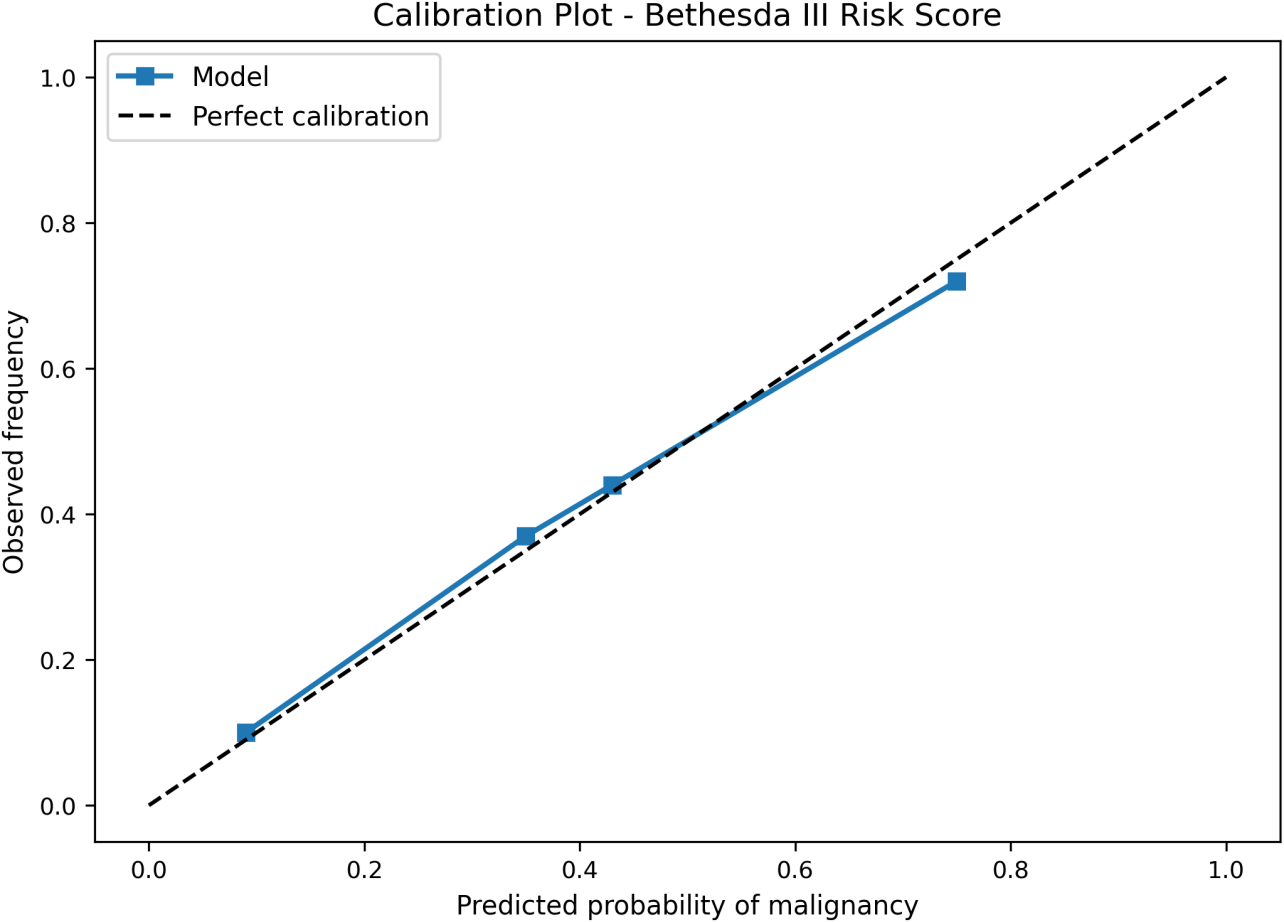
Flow diagram of patient and nodule selection for the analysis.

A total of 114 Bethesda III nodules were identified between 2020–2025 at the four centres. After applying the inclusion and exclusion criteria, 85 patients with one surgically resected Bethesda III nodule each were included in the final analysis (**Figure 1**). Reasons for exclusion: 22 patients were not operated on, and 7 had incomplete records.

### Study population and malignancy rates

A total of 85 Bethesda III thyroid nodules with definitive surgical histology were included in the study. Of these, 65 (76.5%) were benign and 20 (23.5%) were malignant according to the final pathology.

### Histological diagnoses

Among benign nodules, follicular nodular disease was the most frequent diagnosis (41/65, 63.1%), followed by follicular adenoma (17/65, 26.2%). Less common benign diagnoses included NIFTP (1.5%), WDT-UMP (1.5%), lymphocytic thyroiditis (1.5%), oncocytic adenoma (1.5%), Hurthle cell adenoma (1.5%), hyalinising trabecular adenoma (1.5%), and no significant histopathological findings (1.5%). Among malignant nodules, papillary thyroid carcinoma was the predominant histological type (15/20, 75%), followed by follicular carcinoma (3/20, 15%), oncocytic carcinoma (1/20, 5%), and medullary thyroid carcinoma (1/20, 5%). NIFTP and WDT-UMP were coded as benign in accordance with the current WHO Health Organisation 2022 classification and Bethesda 2023 system, as is standard in risk-stratification studies of indeterminate nodules.

Univariate analysis.

The complete results of the univariate analysis are presented in **Table 1**. The cohort was predominantly female (67/85, 78.8%). No sociodemographic, metabolic, or biochemical variable reached statistical significance, although male sex (OR 1.89), age 45–54 years (OR 3.17, *p* = 0.09), and anti-TPO positivity (OR 2.51, *p* = 0.132) showed numerical trends. Among the ultrasonographic features, microcalcifications (OR 12.00; *p* < 0.01), macrocalcifications (OR 4.67; *p* = 0.01), lobulated or irregular margins (OR 16.00; *p* = 0.02), and taller-than-wide shape (OR 4.00; *p* = 0.046) were significantly associated with malignancy. The dichotomised TIRADS grouping (TR 4–5 vs. TR 1-3) showed a strong association (OR 8.21, 95% CI 2.13-54.30; *p* < 0.01), with 90% of the malignant nodules classified as high-risk. Small nodule size (≤20 mm) was also significant (OR 2.98; *p* = 0.04). Nuclear atypia on FNA was the strongest individual predictor (OR 6.37, *p* < 0.01). Lymphocytic thyroiditis on FNA was significantly associated with malignancy (OR 3.83; *p* = 0.01).

### Multivariate logistic regression model

After multivariable logistic regression with backward stepwise elimination, three variables remained independently and significantly associated with malignancy (**Table 2**): moderate-to high-risk ACR-TIRADS (TR 4-5; adjusted OR 6.09, 95% CI 1.45-41.90; *p* = 0.03), nuclear atypia on FNA (adjusted OR 5.20, 95% CI 1.57-18.60; *p* < 0.01), and lymphocytic thyroiditis on FNA (adjusted OR 3.78, 95% CI 1.13-13.43; *p* = 0.03). The model intercept was -3.6942. Although these three variables were identified as independent predictors, the wide 95% confidence intervals (e.g., 1.45-41.90 for TR 4-5) reflect the limited number of malignant events in our cohort, indicating that the exact magnitude of the effect should be interpreted cautiously.

**Table 2:** Multivariable logistic regression model for malignancy prediction in Bethesda III thyroid nodules.

Variable	Beta	Adjusted OR (95% CI)	P-value
TIRADS dichotomised grouping			
Low risk (TR 1-3)	Ref		
Moderate-high risk (TR 4-5)	1.8063	6.09 (1.45-41.90)	0.03
Nuclear atypia on FNA			
No	Ref		
Yes	1.6494	5.20 (1.57-18.60)	<0.01
Lymphocytic thyroiditis on FNA			
No	Ref		
Yes	1.3286	3.78 (1.13-13.43)	0.03
Intercept	-3.6942	NA	<0.01

OR, odds ratio; CI, confidence interval; FNA, fine-needle aspiration; TIRADS, Thyroid Imaging Reporting and Data System.

### Development of a practical risk score

**Figure 2:**
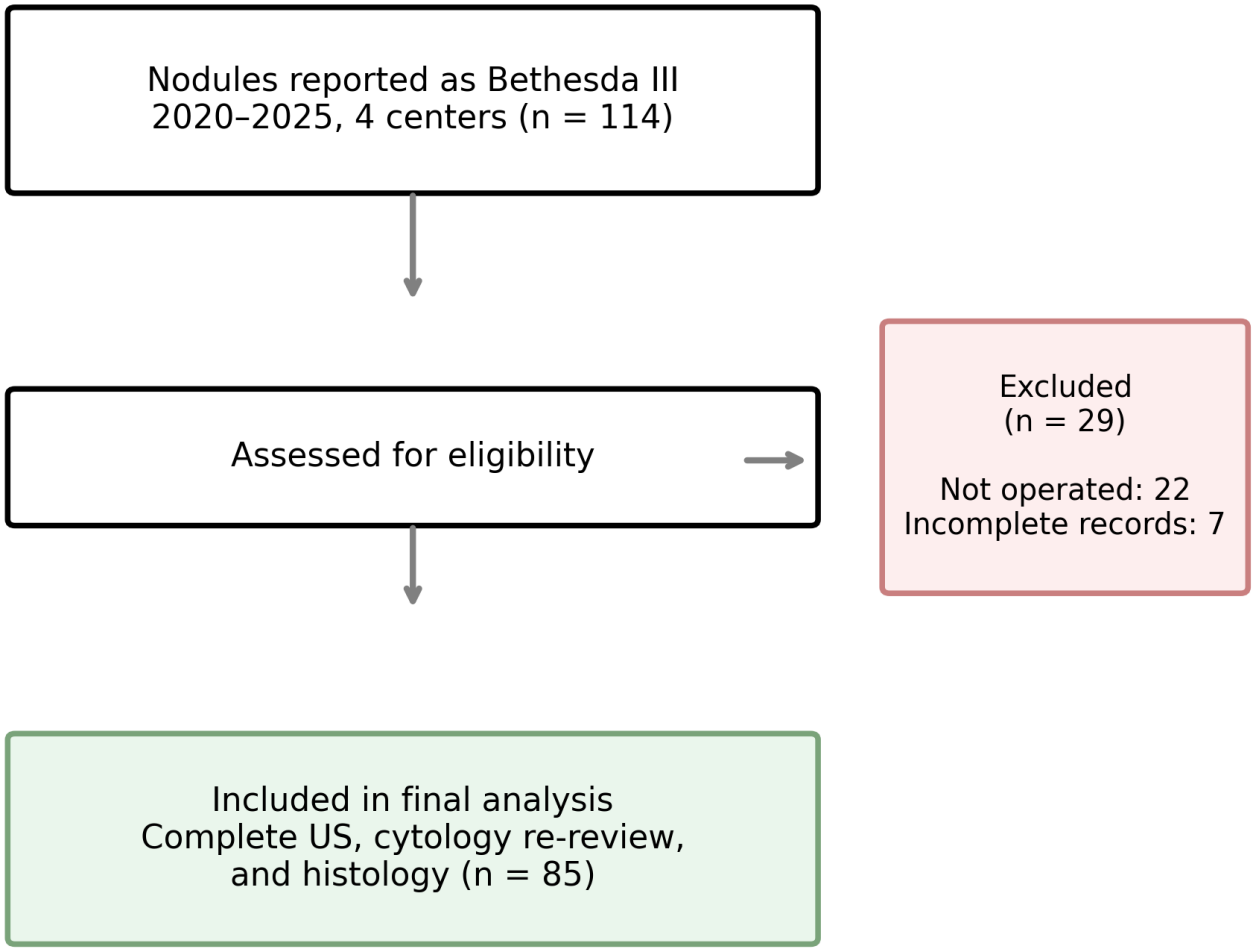
Predicted probability of malignancy according to combinations of the Bethesda III risk score components.

A simplified, integer-based risk score was derived from the beta coefficients of the final multivariable logistic regression model. Integer points were assigned in proportion to the magnitude of the beta coefficients to preserve the relative weight of each predictor in a simplified clinical tool. The score assigned 6 points for moderate-to-high risk TIRADS (TR 4-5), 5 points for nuclear atypia on FNA, and 4 points for lymphocytic thyroiditis on FNA, yielding a total score ranging from 0 to 15 points. **Table 3** presents the eight possible score combinations with corresponding predicted probabilities of malignancy, ranging from 2.4% (score 0) to 74.8% (score 15). To facilitate clinical interpretation, these combinations and their associated malignancy probabilities are graphically summarized in **Figure 2**.

**Table 3:** Risk score combinations and predicted probability of malignancy.

Lymphocytic thyroiditis (FNA)	Nuclear atypia	TIRADS 4-5	Score	Predicted malignancy
No	No	No	0	2.4%
Yes	No	No	4	8.6%
No	Yes	No	5	11.5%
No	No	Yes	6	13.1%
Yes	Yes	No	9	32.8%
Yes	No	Yes	10	36.4%
No	Yes	Yes	11	44.1%
Yes	Yes	Yes	15	74.8%

### Discriminative performance of the risk score

**Figure 3:**
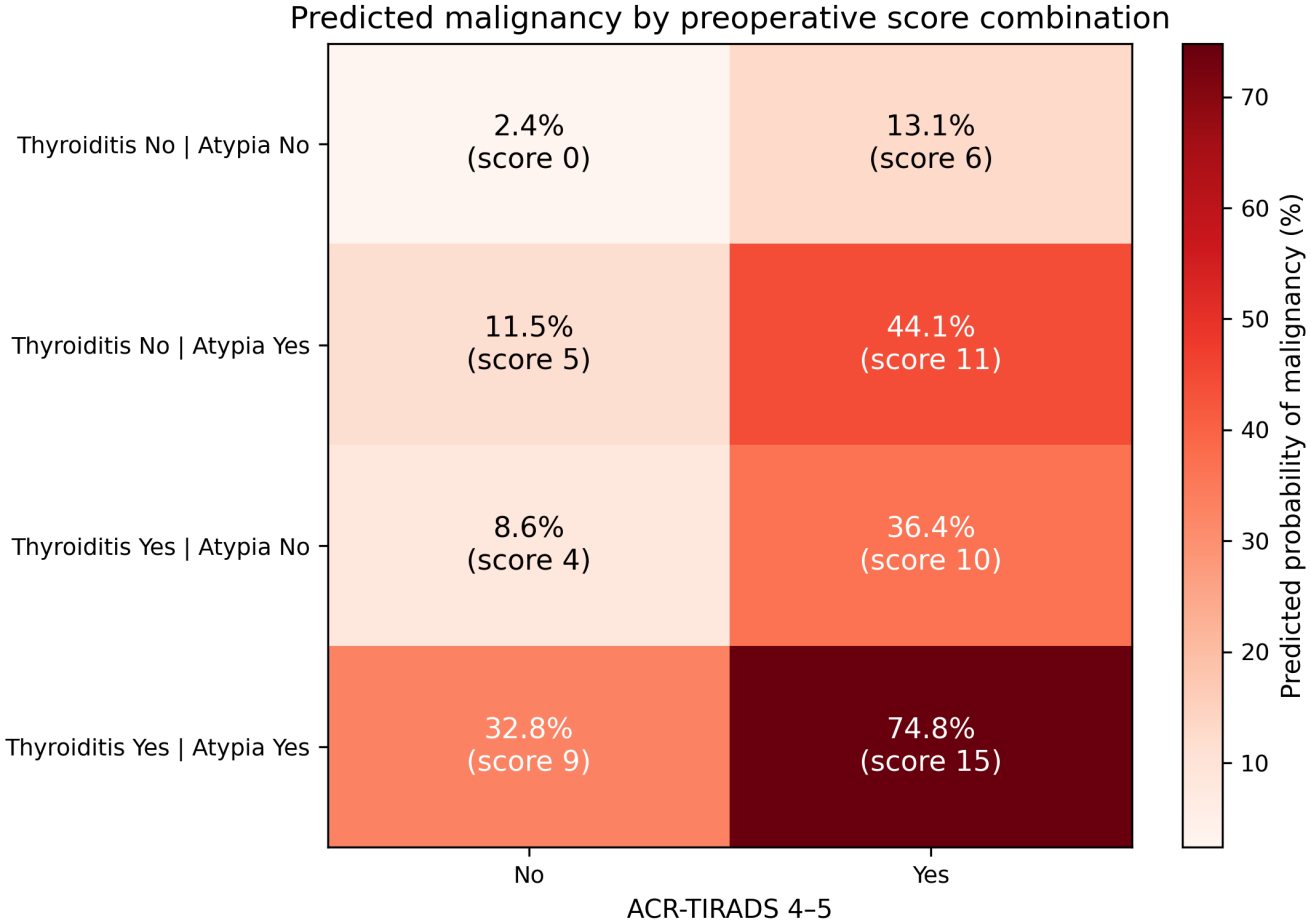
Receiver operating characteristic (ROC) curve of the Bethesda III malignancy risk score.

**Figure 4:**
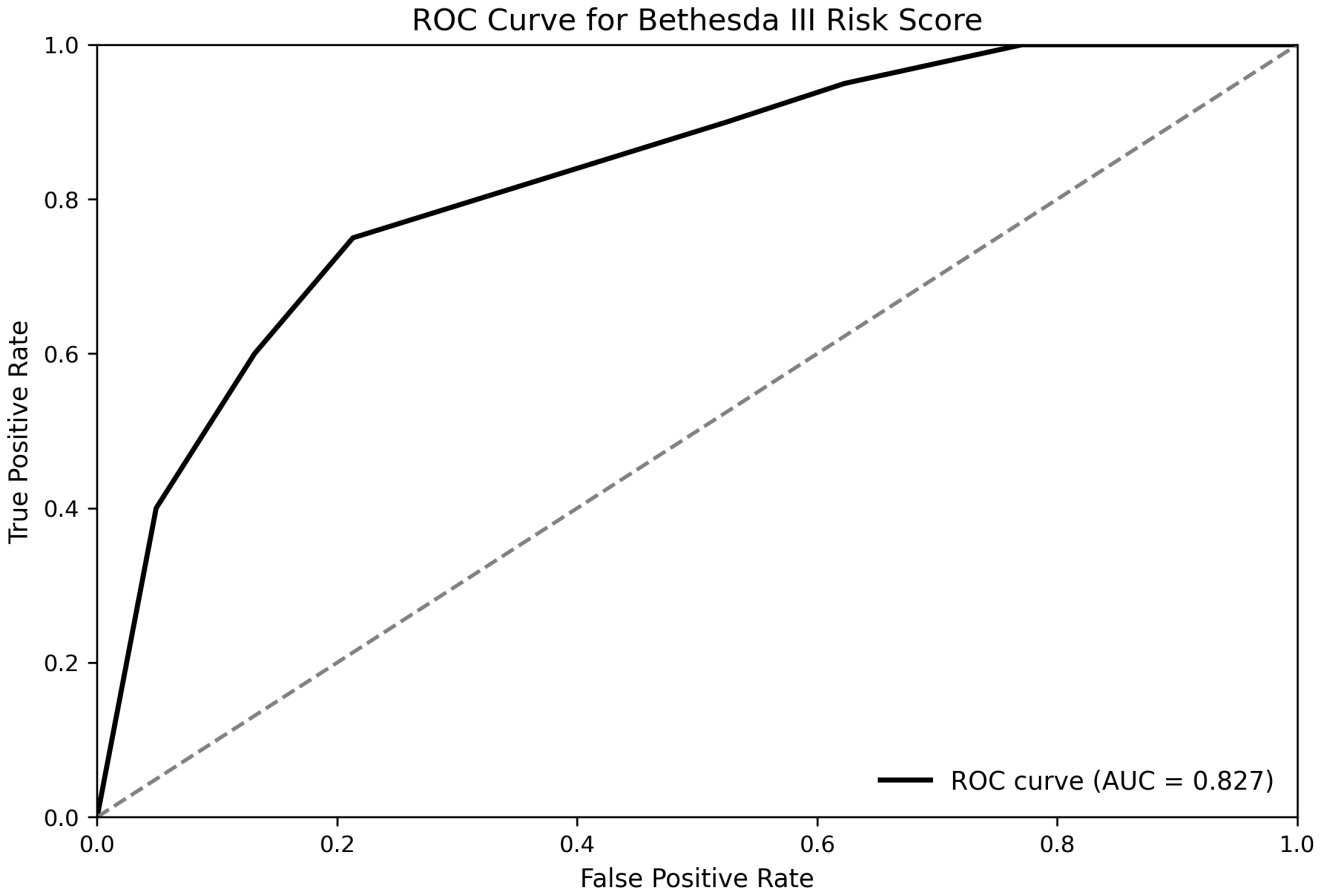
Calibration plot of the Bethesda III malignancy risk score. The solid line represents the observed versus predicted probabilities, and the dashed line indicates perfect calibration. The model showed excellent calibration (optimism-corrected slope 0.91; intercept, 0.03) after 1,000 bootstrap replications.

Receiver operating characteristic (ROC) curve analysis showed good apparent discrimination of the risk score (AUC 0.827) (**Figure 3**). The model also showed an acceptable calibration. The optimism-corrected calibration slope was 0.91 and the intercept was 0.03, suggesting limited overfitting and minimal systematic over- or underestimation of risk. The calibration plot (**Figure 4**) shows an overall agreement between the predicted and observed malignancy probabilities. Internal bootstrap validation using 1,000 resamples showed minimal estimated optimism in the model. The apparent AUC was 0.827, mean optimism was −0.008, and resulting optimism-corrected AUC was 0.836 (95% CI 0.827-0.896) (**Table 4**). Given the relatively small sample size, slightly negative optimism was interpreted as reflecting resampling variability rather than evidence of improved out-of-sample performance.

**Table 4:** Internal validation of the Bethesda III risk score.

Metric	Value
Apparent AUC	0.827
Mean optimism (1,000 bootstraps)	−0.008
Optimism-corrected AUC	0.836 (95% CI 0.827–0.896)
Calibration slope (corrected)	0.91
Calibration intercept (corrected)	0.03

### Optimal score cut-off

The diagnostic performance of the risk score was evaluated across multiple cut-off values (**Table 5**). The optimal cut-off was ≥10 points, which maximised the Youden Index at 0.53, achieving a sensitivity of 75% (15/20 true positives), specificity of 78% (51/65 true negatives), 14 false positives, and 5 false negatives. At this threshold, the positive and negative predictive values 51.7% and 91.1%, respectively, in the derivation cohort. At this threshold, the score identified 15 of 20 malignant nodules and correctly classified 51 of 65 benign ones.

**Table 5:** Diagnostic performance of the risk score at different cut-off values. At cut-off ≥10: 51/65 benign nodules would avoid unnecessary surgery (78% specificity) while missing 5/20 malignancies (25%). At cut-off ≥6: only 2 malignancies missed but 34 benign nodules still operated (48% specificity).

Score cut-off	Sensitivity	Specificity	Youden Index	TP	TN	FP	FN
0	1.00	0.00	0.00	20	0	65	0
4	1.00	0.25	0.25	20	16	49	0
5	0.95	0.38	0.33	19	25	40	1
6	0.90	0.48	0.38	18	31	34	2
**≥10**	**0.75**	**0.78**	**0.53**	**15**	**51**	**14**	**5**
11	0.60	0.88	0.48	12	57	8	8
15	0.40	0.95	0.35	8	62	3	12

Bold values indicate the optimal cut-off point according to the Youden Index. TP, true positives; TN, true negatives; FP, false positives; FN, false negatives.

## Discussion

The management of Bethesda III thyroid nodules remains one of the most debated topics in thyroid disease, as these lesions occupy a diagnostic gray zone that challenges clinicians to balance the risk of missing a malignancy against the burden of unnecessary surgery (11, 12). In this multicentre study of surgically resected Bethesda III nodules, the malignancy rate was 23.5% within the 13-30% range proposed by the 2023 Bethesda System (12) and consistent with previous surgical series, in which the risk of malignancy is generally higher than unselected populations (18, 23).

Multivariable analysis identified three independent predictors of malignancy: moderate-high risk ACR-TIRADS category (TR 4-5), nuclear atypia on FNA, and lymphocytic thyroiditis on FNA. The confirmation of nuclear atypia as a robust predictor is consistent with both institutional series and higher-level evidence. In particular, a systematic review and meta-analysis showed that cytologically indeterminate nodules with nuclear atypia carry a significantly higher risk of malignancy than those without nuclear atypia, supporting the biological and clinical relevance of this distinction (13–15, 24). This finding also agrees with data from a Spanish cohort, in which malignancy was markedly higher in nodules classified as cytological atypia than in those with architectural atypia (14). Indeed, the 2023 Bethesda System formally acknowledged this differential risk by simplifying Bethesda III into two subgroups: AUS with nuclear atypia (AUS-N) and AUS with other atypia (AUS-O) (12, 17).

The ultrasound component also played a relevant role in risk stratification. The dichotomised ACR-TIRADS grouping showed the strongest association in the model, and most malignant nodules were classified as moderate- or high-risk on ultrasound. These results are in line with previous studies showing a significant association between moderate-to-high-risk ACR-TIRADS categories and malignancy in Bethesda III nodules (20, 21, 24, 25). Beyond formal categorization, critical or expert ultrasound reassessment may further refine decision-making, particularly when suspicious features such as microcalcifications or irregular margins are present (24, 25). Therefore, the value of the ultrasound component in the score should be understood as part of a broader ultrasound-cytology integration process, rather than merely as a static classification label.

A practical contribution of this study is the translation of the regression model into a simple integer-based score. Assigning 6 points for TIRADS 4-5, 5 points for nuclear atypia, and 4 points for lymphocytic thyroiditis on FNA generated eight possible combinations, with estimated malignancy probabilities ranging from 2.4% to 74.8%. The ≥10-point cut-off provided the best overall balance between sensitivity and specificity according to the Youden Index. However, these weights should be interpreted as an exploratory approximation rather than definitive values, as they were derived from a limited cohort and may vary in independent populations. Accordingly, although the score showed good apparent discrimination and acceptable internal performance after bootstrap validation, its clinical usefulness requires external confirmation before systematic implementation can be considered.

From a clinical standpoint, the score could be positioned early in the diagnostic pathway, after the initial Bethesda III cytology and baseline ultrasound assessment, and before deciding on repeat FNA, molecular testing, or surgery. Its purpose would not be to replace these strategies, but to serve as a triage tool to guide which patients may benefit most from each subsequent step. This approach is consistent with evidence supporting the combined use of repeat FNA and ultrasound-based risk stratification, as well as with current recommendations favouring risk-adapted, stepwise decision-making in thyroid nodule management (5, 21).

The interpretation of the cut-off should remain cautious. Although ≥10 points was the statistically optimal threshold in this cohort, a lower cut-off, such as ≥6 points, may be preferable in clinical settings where minimising false negatives is prioritised, even at the cost of reduced specificity.

This distinction may be particularly relevant in Bethesda III nodules, where the main clinical concern is often not to miss malignancy, especially when repeat FNA, molecular testing, or surgery remain available as second-line strategies.

In practical terms, the score may help organise management into broad, risk-oriented categories. Scores of 0–5 points, corresponding to lower estimated malignancy probabilities, may support ultrasound surveillance in the absence of additional worrisome features. Scores of 6–9 points would identify an intermediate-risk group in whom management should be individualised according to ultrasound pattern, nodule growth, patient preference, local expertise, and availability of molecular testing. Scores of ≥10 points may define a higher-risk subgroup in which earlier surgical consideration or additional molecular evaluation could be justified. Nevertheless, low scores should not be interpreted as excluding malignancy, particularly in AUS-other nodules and non-papillary thyroid malignancies, such as follicular, oncocytic, or medullary carcinoma, in which nuclear atypia may be less prominent on cytology. In such scenarios, discordant sonographic, clinical, or biochemical findings should prevail over the score alone.

The independent association between lymphocytic thyroiditis on FNA and malignancy is one of the most distinctive aspects of this study. Although the relationship between chronic lymphocytic thyroiditis or Hashimoto thyroiditis and thyroid cancer has been debated for decades, this variable remained significant in our model after adjustment for nuclear atypia. However, given the retrospective design and limited sample size, this finding should be interpreted as hypothesis-generating rather than as definitive evidence of causality or stable incremental risk. Chronic inflammation has been linked to mechanisms potentially involved in malignant transformation, particularly papillary carcinoma, through shared molecular pathways, oxidative stress, and dysregulated immune responses (26). Our results differ partially from some previous reports and add exploratory evidence that lymphocytic thyroiditis on FNA may have value in risk stratification within a surgically selected cohort (22, 26). The clinical relevance of lymphocytic thyroiditis may extend beyond its association with malignancy. In surgical series of AUS/FLUS nodules, concomitant chronic lymphocytic thyroiditis appears to limit the discriminatory value of molecular testing, suggesting that an inflammatory background may complicate interpretation or incremental utility of these assays (27, 28). Thus, including this routinely available cytological variable may provide a practical advantage over more complex models and help identify patients in whom clinical decision-making may rely particularly on repeat FNA and careful ultrasound-cytology integration, rather than on indiscriminate escalation to molecular testing.

Univariate analysis also identified individual ultrasound features associated with malignancy, including microcalcifications, macrocalcifications, taller-than-wide shape, irregular margins, and nodule size ≤20 mm. In the multivariable model, however, these findings were integrated into the dichotomised ACR-TIRADS variable, which summarises multiple sonographic characteristics into a single composite predictor. The histological profile of malignant nodules was similar to that reported in the literature, with papillary thyroid carcinoma predominating. Among benign diagnoses, follicular nodular disease and follicular adenoma were the most frequent, reflecting the high proportion of benign histology in surgically managed Bethesda III nodules and reinforcing the need for improved preoperative risk stratification (11, 12, 18).

Overall, the score should be regarded as a complementary triage tool rather than an alternative to molecular testing. Its main potential value may lie in selecting Bethesda III nodules in which further diagnostic escalation is justified, thereby reserving molecular assays for cases in which their expected incremental value is greatest, particularly intermediate-risk nodules after repeat FNA or expert ultrasound reassessment (27, 29). Beyond confirming the established predictive role of nuclear atypia and sonographic suspicion, the main novelty of this study lies in translating these findings into a simple integer-based preoperative score and in identifying lymphocytic thyroiditis on FNA as a potential independent contributor to risk stratification.

## Limitations

This study has several limitations that should be considered when interpreting the findings. First, its retrospective, multicentre design and the fact that only surgically resected nodules were included introduced significant selection and verification biases. The observed malignancy rate (23.5%) is therefore higher than that in the overall Bethesda III population, as nodules perceived to be at lower risk are typically managed conservatively. Consequently, the risk score was calibrated exclusively for a surgical cohort, and its performance in non-operated patients remains unknown. Notably, 22 patients with Bethesda III nodules were managed conservatively without surgery during the study period. While detailed follow-up data for this group fell outside our surgical inclusion criteria, their exclusion likely contributes to the relatively high baseline malignancy rate (23.5%) observed in our surgical cohort, underscoring the selection bias inherent to this study design.

Second, the relatively small sample size (n = 85, with only 20 malignant events) limits the statistical power and increases the risk of model instability. Although model complexity was deliberately restricted to three predictors and internal bootstrap validation suggested limited optimism (mean optimism −0.008; optimism-corrected AUC 0.836), the events-per-variable ratio (≈6.7) remained at the lower boundary of commonly accepted rules of thumb. In addition, the slightly negative optimism estimate is unusual and was interpreted as likely reflecting resampling variability related to the limited sample size rather than a true improvement in model performance. Therefore, some degree of overfitting cannot be completely ruled out, and external validation is essential in this regard. The events-per-variable ratio (≈6.7) remained at the lower boundary of commonly accepted rules of thumb. Consequently, the regression coefficients—and the resulting point values in our score—are susceptible to sample-specific variations. External validation is strictly required to confirm whether this specific weighting remains optimal across different clinical settings.

Third, although the study was multicentre, both ultrasound (ACR-TIRADS) and cytological assessments were performed by multiple radiologists and pathologists without a centralized review, which may have introduced interobserver variability. In addition, the possibility of residual confounding cannot be excluded, as unmeasured variables (e.g. molecular markers, family history, or nodule growth kinetics) were not available.

Finally, papillary thyroid carcinoma constituted 75% of malignancies; therefore, the score may be better calibrated for tumours with nuclear features than for follicular, oncocytic, or medullary carcinomas, in which nuclear atypia is often less prominent on cytology.

Taken together, these limitations indicate that the proposed risk score should be regarded as an exploratory tool. Prospective, large-scale studies with external validation in both surgical and non-surgical cohorts are required before it can be recommended for routine clinical use.

In conclusion, in this multicentre cohort of surgically resected Bethesda III thyroid nodules, moderate-to-high ultrasound risk (ACR-TIRADS 4-5), nuclear atypia on FNA, and lymphocytic thyroiditis on FNA were independently associated with malignancy. These routinely available preoperative variables were used to derive a simple, three-item exploratory risk score. The score showed preliminary discriminatory ability in the derivation cohort and may support preoperative risk stratification by helping to identify lower- and higher-risk subsets of Bethesda III nodules. Further validation in larger, independent, and non-surgical cohorts is required before routine clinical application.

## Data Availability

The raw data supporting the conclusions of this article are not publicly available due to patient confidentiality and data protection restrictions. De-identified data may be made available by the corresponding author upon reasonable request, subject to applicable ethical and institutional approvals.
